# The Differential Diagnosis of Discrepant Thyroid Function Tests: Insistent Pitfalls and Updated Flow-Chart Based on a Long-Standing Experience

**DOI:** 10.3389/fendo.2020.00432

**Published:** 2020-07-07

**Authors:** Irene Campi, Danila Covelli, Carla Moran, Laura Fugazzola, Chiara Cacciatore, Fabio Orlandi, Gabriella Gallone, Krishna Chatterjee, Paolo Beck-Peccoz, Luca Persani

**Affiliations:** ^1^Department of Endocrine and Metabolic Diseases, Istituto Auxologico Italiano, Istituto Di Ricovero e Cura a Carattere Scientifico (IRCCS), Milan, Italy; ^2^Department of Pathophysiology and Transplantation, University of Milan, Milan, Italy; ^3^Endocrinology Unit, Graves' Orbitopathy Center, Fondazione Cà Granda Istituto Di Ricovero e Cura a Carattere Scientifico, Milan, Italy; ^4^Metabolic Research Laboratories, Wellcome Trust-MRC Institute of Metabolic Science, University of Cambridge and National Institute for Health Research Cambridge Biomedical Research Center, Addenbrooke's Hospital, Cambridge, United Kingdom; ^5^Section of Endocrinology, Department of Oncology, Humanitas-Gradenigo Hospital, University of Turin, Turin, Italy; ^6^Endocrinology Unit, Sedes Sapientiae Hospital, Turin, Italy; ^7^Department of Clinical Sciences and Community Health, University of Milan, Milan, Italy

**Keywords:** hyperthyroidism, pituitary adenoma, resistance to thyroid hormone, thyroid hormone, thyrotropin (TSH), immuno-assay

## Abstract

**Background:** Discrepant thyroid function tests (TFTs) are typical of inappropriate secretion of TSH (IST), a rare entity encompassing TSH-secreting adenomas (TSHoma) and Resistance to Thyroid Hormone (RTHβ) due to *THRB* mutations. The differential diagnosis remains a clinical challenge in most of the cases. The objective of this study was to share our experience with patients presenting with discrepant TFTs outlining the main pitfalls in the differential diagnosis.

**Methods:** medical records of 100 subjects with discrepant TFTs referred to Thyroid Endocrine Centers at the University of Milan were analyzed, retrospectively. Patients were studied by dynamic testing (TRH test, T3-suppression test, or a short course of long-acting somatostatin analog, when appropriate), *THRB* sequencing, and pituitary imaging.

**Results:** 88 patients were correctly diagnosed as RTHβ with (*n* = 59; 16 men, 43 women) or without *THRB* variants (*n* = 6; 2 men, 4 female) or TSHoma (*n* = 23; 9 men, 14 women). We identified 14 representative subjects with an atypical presentation or who were misdiagnosed. Seven patients, with spurious hyperthyroxinemia due to assays interference were erroneously classified as RTHβ (*n* = 4) or TSHoma (*n* = 3). Three patients with genuine TSHomas were classified as laboratory artifact (*n* = 2) or RTHβ (*n* = 1). Two TSHomas presented atypically due to coexistent primary thyroid diseases. In one RTHβ a drug-induced thyroid dysfunction was primarily assumed. These patients experienced a mean diagnostic delay of 26 ± 14 months. Analysis of the investigations which can differentiate between TSHoma and RTHβ showed highest accuracy for the T3-suppression test (100% specificity with a cut-off of TSH <0.11 μUI/ml). Pituitary MRI was negative in 6/26 TSHomas, while 11/45 RTHβ patients had small pituitary lesions, leading to unnecessary surgery in one case.

**Conclusions:** Diagnostic delay and inappropriate treatments still occur in too many cases with discrepant TFTs suggestive of central hyperthyroidism. The insistent pitfalls lead to a significant waste of resources. We propose a revised flow-chart for the differential diagnosis.

## Introduction

TSH secreting pituitary adenomas (TSHomas) and Resistance to Thyroid Hormone due to mutations in the *THRB* gene (RTHβ) are two possible underlying causes of the rare clinical entity of inappropriate secretion of TSH (IST), which is characterized by hyperthyroxinemia and non-suppressed TSH levels.

The differential diagnosis of IST is often challenging (1). Major factors hampering diagnosis are the low incidence of the condition, insufficient awareness or experience of diagnosis and management, and absence of diagnostic tests with high sensitivity or specificity (2–4).

Laboratory artifacts resulting in discrepant thyroid function tests (TFTs) may arise due to anti-T4 or heterophile antibodies (5) or abnormal concentration or affinity of TH transport proteins (TBG, albumin, and transthyretin) (6); these conditions are significantly more frequent than genuine IST, and as such, result in real potential for misdiagnosis (7). The coexistence of a primary thyroid disorder is frequent, particularly in areas of mild/moderate iodine deficiency (8) and might cause additional diagnostic uncertainty (9, 10). Finally, once a diagnosis of IST is confirmed, TSHomas must be differentiated from RTHβ, due to different management and therapy of these disorders (1).

Several tests and strategies for the differential diagnosis of TSHomas have been proposed, and include biochemical parameters (e.g., SHBG, markers of bone metabolism, serum alpha subunit) (11–13), dynamic testing (e.g., TRH stimulation or T3 suppression tests) (14–19) or a short course of long-acting somatostatin analog (LAR–SMS) administration (20), in addition to pituitary MRI scan and *THRB* sequencing (1). However, several of these investigations are not sufficiently discriminatory, or are available only in some centers; in addition they may be contra-indicated in some cases, or be overly cumbersome and expensive to perform. Thus the differential diagnosis of discrepant TFTs remains a clinical challenge.

Our study aims to share our experience of patients presenting with discrepant TFTs outlining the main pitfalls in the differential diagnosis of IST. In particular, we highlight 14 particular cases with either an atypical clinical presentation or which exemplify consequences of misdiagnosis. Our study shows that an excessive number of patients still experience delayed diagnosis or inappropriate and harmful treatment, with attendant waste of resources and care costs.

## Materials and Methods

### Study Protocol

Medical records of 121 subjects, referred to Endocrine Centers at the University of Milan, in the period 1997–2018 for investigation of IST were analyzed, retrospectively.

We considered as the gold standard for diagnosis of RTHβ the identification of *THRB* mutation located in the three hotspots of the ligand binding domain of the receptor. The absence of a *THRB* mutation despite a clear RTHβ phenotype led to a classification of non-TH RTHβ (21).

The diagnosis of a TSHoma depended upon the combination of several findings, because the frequency of incidental pituitary lesions rendered often inadequate the association of IST and a positive pituitary imaging. Confirmations of a TSHoma were the remission of biochemical alterations and clinical manifestations after pituitary surgery or radiotherapy, or during medical treatment with somatostatin analogs. This latter condition was also accepted as a proof of TSHoma as TFTs can be only transiently affected by the acute administration of somatostatin in RTHβ, but no normalization or even decreases of circulating free thyroid hormones can be seen in RTHβ patients on chronic somatostatin analogs (1, 20).

**Figure 1:**
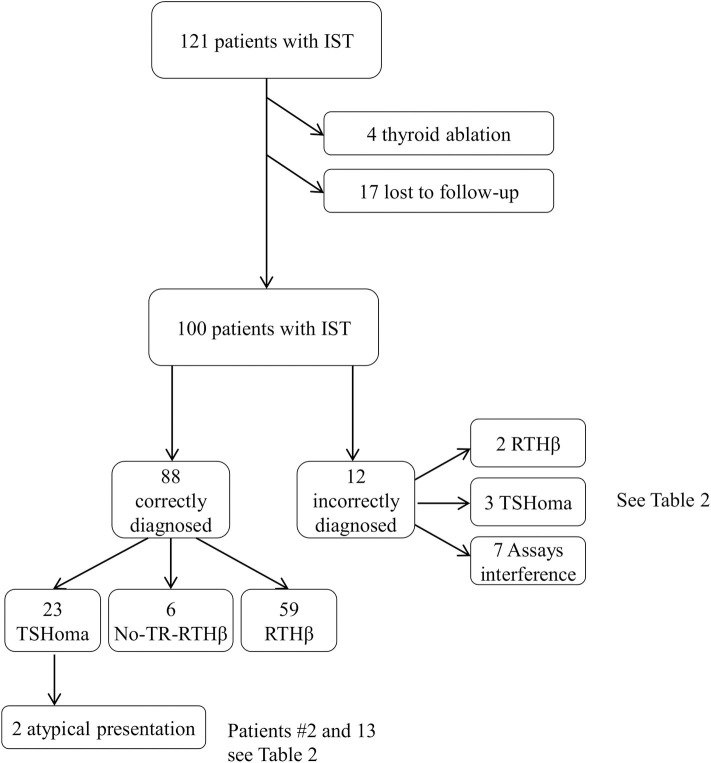
Selection process of the medical record included in this survey. Of the 121 files screened, 100 cases were included in the final analysis. See also Table 2 for further details.

We excluded from the analysis of dynamic testing RTHβ patients previously submitted to thyroidectomy or 131-I radiometabolic therapy (*n* = 4). We also excluded patients with RTHβ without mutations in the *THRB* gene (non-TR-RTHβ) (21) who were lost to follow-up (*n* = 17), although their initial investigations were consistent with RTH and not significantly different compared with patients harboring dominant negative variants in the *THRB* gene (Figure 1).

RTHβ was ultimately diagnosed in sixty-one patients (16 men, 45 women), a non-TR-RTHβ in six, and a TSHoma in 26 cases (9 men, 17 women; 8 macroadenomas, and 18 microadenomas). The remaining seven patients had spurious hyperthyroxinaemia due to interference in thyroid hormone measurement (Figure 1). The 6 patients with non-TR-RTHβ (21) were tested by TRH test, T3 suppression test, and pituitary MRI at diagnosis and two or three times during follow-up (range 7–12 years). Because these patients have a different and unknown molecular cause of peripheral resistance, their response to dynamic testing was not considered for the definition of the proposed cut-offs.

Imaging data and dynamic testing of patients with genuine RTHβ due to *THRB* gene mutations and TSHoma were compared to a control group of 105 controls (36 males, 69 females). The controls were recruited in different ways: (a) volunteers among the personnel of the involved clinical and laboratory units; (b) subjects demonstrated to have normal thyroid function tests after retesting at our Laboratory; (c) patients presenting with unconfirmed hyperprolactinemia; (d) incidental pituitary microlesions <5 mm that were not confirmed at targeted radiological examinations or were found to be non-functional at biochemical testing and upon follow-up of at least 7 years. These subjects gave informed consent for testing and publication of anonymized data for research purposes.

A portion of controls had euthyroid autoimmune thyroid disease (AITD) (33%). These were included in the control group in order to obtain a correct matching since 20 and 32% of RTHβ and TSHoma, respectively, had an associated AITD (Table 1). None of the patients was taking anti-thyroid drugs.

**Table 1:** Clinical characteristics of patients and controls included in the study.

	**Controls**	**TSHomas**	**RTHβ**	**P**
N	105	26	61	
Males/females (*n*)	36/69	9/17	16/45	NS
AITD (*n*)	31	5	21	NS
Age (years)	39 ± 15	42 ± 13	34 ± 15	<0.05^*^; NS^#^^§^
Baseline TSH (μUI/ml)	**2.3 ± 1.6**	3.5 ± 2.6	2.4 ± 2.0	NS^*^; <0.05^#^^§^
Baseline FT4 (pmol/l)	12.6 ± 2.8	**28.1** ± **6.7**	**32.2** ± **12.2**	<0.05^*^^#^; NS^§^
Baseline FT3 (pmol/l)	5.4 ± 1.2	**11.1** ± **3.8**	**10.1** ± **4.3**	<0.05^*^^#^; NS^§^
Expansive lesions at pituitary MRI	**4**/25	**20**/26	**11**/45	<0.001^#^^§^; NS^*^
TRH test (*n*)	**103**	**24**	**61**	
TSH peak (μUI/ml)	16.7 ± 12.4	7.7 ± 7.2	19.0 ± 13.1	<0.001^#^^§^; NS^*^
TSH fold increase	7.8 ± 3.3	**2.3 ± 1.6**	9.3 ± 4.9	<0.001^#^^§^^*^
delta increase TSH (μUI/ml)	14.4 ± 11.2	3.9 ± 5.6	16.5 ± 11.5	<0.001^#^^§^; NS^*^
T3 suppression (*n*)	15	19	16	
TSH (μUI/ml) at day 10	0.02 ± 0.02	**1.9 ± 1.5**	0.3 ± 0.5	<0.0001^#^^§^; NS^*^

*Data are expressed as mean ± SD; abnormal values are indicated in bold*.

* *Between RTHβ and controls*;

§ *between RTHβ and TSHoma*;

# *between TSHoma and controls. IST, inappropriate secretion of TSH; FDH, Familial Dysalbuminemic Hypothyroxinemia; MRI, magnetic resonance imaging; AITD, autoimmune thyroid disorders. Test: one way ANOVA, chi-square test*.

*Reference ranges: TSH 0.3–5.1 mU/L; FT4 10–20 pmol/L; FT3 4.2–7.5 pmol/L*.

From these records, we have highlighted 14 representative cases either with an atypical clinical presentation or misdiagnosed. Eleven patients were females and three were males; their mean age ± SD was 39 ± 16 years (range 19–72 years), with a mean ± SD duration of clinical assessments of 26 ± 14 months (range 6–60).

### Dynamic Tests

Dynamic tests were performed as previously reported (14–20). TRH test was performed by administering 200 μg of TRH intravenously and assessing TSH at baseline, 20, 30, and 60 min after TRH injection. TSH response was defined as the highest TSH peak after TRH (which occurred in all subjects between 20 and 30 min after TRH). The TSH fold increase was calculated as the ratio between the TSH peak and baseline TSH (14). This test was performed in 98, 100, and 90% of controls, RTHβ, and TSHoma, respectively.

T3 suppression test was performed administering fixed doses of 100 μg of sodium liothyronine divided in three doses of 40 + 20 + 40 μg (every 8 h) given by mouth for 10 days (18). TSH was measured at baseline and 2 h after the last dose of T3. Sixty mg/day of propranolol were given in case of heart rate >110 beats/min. The T3 suppression test was performed in 14, 26, and 70% of controls, RTHβ, and TSHoma, respectively. In most of these cases the TRH test, as described above, was repeated after T3 suppression and TRH was injected 2 h after the last T3 dose.

For the LAR-SMS test 30 mg of the somatostatin analog was administered every 28 days for 2–3 months in 13 TSHoma and 4 RTHβ. TSH FT4 and FT3 were measured at baseline, before each injection and 28 days after the last one (20).

Thyroid function was assayed on the two-step immunoassay DELFIA platform (PerkinElmer Turku, Finland) in the majority of the cases and on Elecsys 2010 and Cobas 6000 analyzer (Roche, Basil, Switzerland) in the remaining 20% of cases. Since during the study period both these assays underwent upgrading of the methods by the manufacturer, the cross comparison of FT4 and FT3 (expressed in pmol/L) levels was undertaken after expressing them as a percentage of the upper limit of the assay normal range.

MRI was performed in 20, 70, and 100% of controls, RTHβ, and TSHoma, respectively.

## Results

### TRH Stimulation and T3 Suppression Tests

Both TSH peak and fold increase response to TRH were blunted in TSHoma patients compared with RTHβ or controls (*p* < 0.001), whilst RTHβ cases showed a greater fold increase in TSH compared with controls (*p* < 0.001; Table 1). We obtained comparable results following exclusion of RTHβ and TSHoma patients with high baseline TSH levels (data not shown).

**Figure 2:**
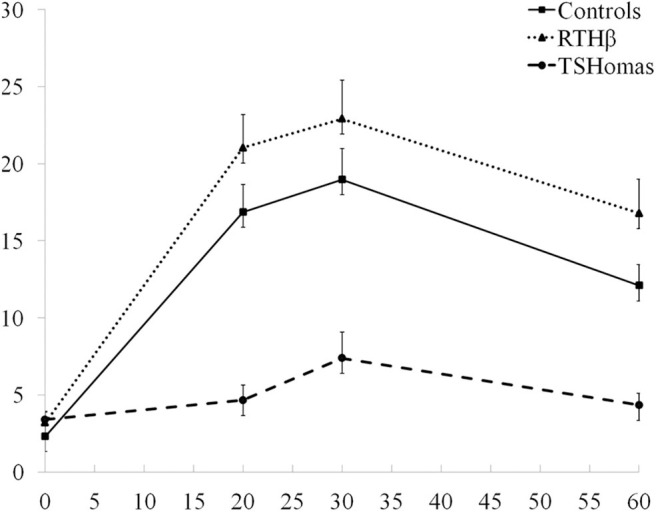
TRH stimulation test results. The three curves represent the mean TSH values ± SEM at baseline, 20, 30, and 60 min after TRH injection in TSHoma and RTHβ patients and controls.

The TSH response curves following TRH stimulation in controls, RTHβ and TSHomas are summarized in Figure 2. With the TSH fold increase to TRH being similar in men and women with TSHoma or RTHβ (not shown), we did not identify a gender-specific TSH cut-off.

From ROC curve analysis of TSH fold increase, we identified a cutoff of >5.2 (92% sensitivity; 96.2% specificity) that differentiates TSHomas from RTHβ (not shown).

TSH levels after T3 suppression were higher in TSHoma (1.9 ± 1.5 mean ± SD) than in RTHβ (0.3 ± 0.5; *p* < 0.001) or controls (0.02 ± 0.02; *p* < 0.0001), while in RTHβ were not statistically different than in controls. ROC analysis identified TSH cut-off values post T3 suppression of <0.11 μUI/ml (not shown). This value allowed 100% specificity (each TSHoma had a TSH > 0.11), with all controls being correctly identified as not having a TSHoma.

**Figure 3:**
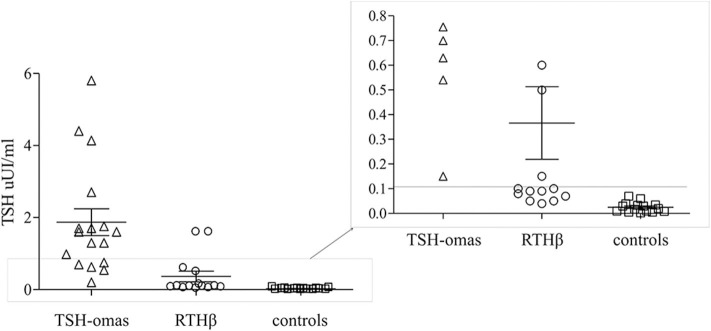
T3 suppression test. Scatter plot of the TSH values after T3 administration for 10 days in TSHomas, genetically-proven RTHβ and controls. In the right panel, the detail of patients with RTH with TSH values were overlapping those of TSH-oma patients.

However, after T3 administration we found overlapping TSH values between 5 RTHβ patients and the TSHomas (Figure 3).

### Other Investigations

In most cases, we performed a TRH test after T3 administration. In controls, we found a complete suppression of TSH response. Most RTHβ patients exhibited a partially suppressed TSH response; however, two RTHβ cases (carrying the *THRB* variants p.R429Q and p.Y321C) who showed incomplete suppression of baseline TSH levels (0.6 and 0.5 μIU/ml, respectively), maintained a significant TSH response to TRH (5.3 and 3.9, respectively). In 100% of the TSHoma patients, baseline TSH levels did not suppress after T3 but retained response to TRH in about 15% of cases.

Thirteen TSHoma and four RTHβ patients underwent a short course of LAR-SMS injections (30 mg/28 days for 2 months). In TSHoma, the average reduction in FT3 and FT4 levels was 40.1 ± 2 and 46.3 ± 24% (mean ± SD), while in RTHβ it was 11.7 ± 4 and 7.2 ± 6%, respectively. Two TSHomas were resistant to LAR-SMS.

Pituitary MRI showed abnormalities compatible with a microadenoma in 11/45 RTHβ and in 3/7 patients with spurious hyperthyroxinemia whereas a pituitary microadenoma was not visualized initially in 6/26 TSHomas (Table 1).

Alpha glycoprotein subunit (αGSU) was measured by radioimmunoassay in 14/26 TSHoma and 12/61 RTHβ cases, with calculation of TSH/αGSU molar ratio. In 11/14 TSHoma patients (79%), we found an elevated molar ratio (>1), but the molar ratio was normal in 3/14 patients with micro-TSHoma and increased in five postmenopausal RTHβ women.

The 66% of RTHβ patients were familial case, however in 30% of them the thyroid function of the relatives was not performed prior to referral.

## Series of Challenging Patients

Patient #1: (i) Referral diagnosis, assay interference; at baseline a multinodular goiter was diagnosed and a cytological analysis of a 20 mm right sided thyroid nodule ruled out malignancy. The patient's earliest tests showed discrepant TFTs (TSH 3.93 μUI/ml RR 0.28-4.8; FT4 21.6 pmol/l RR 10-20; FT3 7.6 pmol/l RR 2.9–7), but results were dissimilar when repeated in a different laboratory (TSH 4.1 μUI/ml RR 0.28–4.8; FT4 16 pmol/l RR 10–20 FT3 5.9 pmol/l RR 2.9–7), suggesting assay interference. Levothyroxine (L-T4) therapy was introduced aiming to reduce the goiter volume. Despite the lack of TSH suppression, a 50% reduction of the nodule was seen but accompanied by a progressive rise of FT4 and FT3. Nevertheless, this was interpreted as overtreatment and L-T4 was discontinued on July 2007. During 3-years follow-up, FT4 and FT3 normalized. Later, the patient underwent surgery for renal cell carcinoma and endocrine evaluation was deferred for 2 years.

(ii) Interventions: At that time the IST became evident and persistent, assay interference was definitely excluded by a two-step method for TH determination. TSH levels did not increase after TRH stimulation and MRI scan showed a pituitary macroadenoma extending into the right suprasellar cistern (Table 2). Increased PRL levels (110.1 ng/ml, reference range (RR 3–20) were also found. LAR-SMS was started, as the patient refused surgery. Thyroid function and PRL levels normalized, suggesting a TSH/PRL cosecreting macroadenoma, while the dimension of the adenoma showed only a small change over 5-years of follow-up (12 and 10 mm in 2012 and 2017, respectively). The reason for normalization of PRL levels is unclear (22, 23), but this phenomenon is more likely to occur in mixed adenomas. (iii) Ultimate diagnosis, mixed PRL/TSHoma.

**Table 2:** Clinical characteristic of the 14 patients included in the case series.

**Patient**	**#1**	**#2**	**#3**	**#4**	**#5**	**#6**	**#7**	**#8**	**#9**	**#10**	**#11**	**#12**	**#13**	**#14**
Age (range)	60–65	70–75	18–25	18–25	36–40	18–25	40–45	30–35	30–35	50–55	40–45	50–55	30–35	26–30
Referral diagnosis	interf.	IST	TSHoma	RTHβ	interf	TSHoma	RTHβ	TSHoma	RTHβ	RTHβ	RTHβ	TSH-oma + GD	thyroiditis	interf
Ultimate diagnosis	TSHoma	TSHoma + ATA	interf	interf	TSHoma + GD	RTHβ	TSHoma	interf	interf	interf	interf	interf	TSHoma	RTHβ
diagnostic delay (months)	36	12	24	24	60	36	24	36	24	24	12	6	15	28
TSH uUI/ml	5.65	1.2	2.1	2.77	1.290	1.3	3.1	1.25	0.81	2.8	2.43	0.57	2.75	1.58
FT4 pmol/L	24.5	25.6	48.7	23.1	23.3	35.3	41.6	34.4	23.2	36.1	22.4	62.5	17.6	26.8
FT3pmol/L	6.3	11.8	17.7	7.3	7.3	8.13	23	12.5	9.5	14.3	10.1	11.5	8.0	6.9
SHBG nmol/L	76^*^	-	29.7^**^	31^**^	162.0^*^	NA	166^*^	80^*^	131^*^	NA	74^*^	77^*^	–	
TRH test (TSH peak)	7.84	2.5	7.32	9.7	1.7	10.3	27.3	11.8	8.7	18.8	20.9	NA	4.55	21.5
T3 test (TSH)	–	–	–	0.005	1.3	–	2.7	0.001	0.005	–	–	–	1.76	–
TSH uUI/ml LAR^*^	2.86	0.84	1.6	1.4	0.025	–	1.2	–	–	–	–	–	–	–
FT4 pmol/L LAR^*^	10.1	19.7	34.4	17.6	20.9	–	16.1	–	–	–	–	–	–	–
FT3 pmol/L LAR^*^	3.7	7.4	13.2	3.8	6.6	–	6.4	–	–	–	–	–	–	–
Thyoid	NG	NG	NG	AITD	G, AITD	G	NG	Normal	Normal	Normal	Normal	NG	NG	AITD
Pituitary MRI	12 mm	8 mm	3 mm	3 mm	no	3 mm	4 mm	no	7 mm	no	no	NA	2 mm	3 mm cyst
THRB gene	wt	wt	wt	wt	wt	R429Q	wt	wt	wt	wt	wt	NA	NA	M310V
Comorbidities	RCC	PAF	DMD	Lipodystrophy, hyper-insulinemia	GD	none	none	HT	HT	HT, PAF MGUS	HT	MGUS	–	Bipolar disorder
TSS (Y/N)	N	Y	N	–	N	Y	Y	–	–	–	–	–	Y	N
Histology	–	TSHoma	–	–	–	Normal pituitary	Pituitary adenoma	–	–	–	–	–	TSHoma	–
Cured	–	Y	–	–	–	–	Y	–	–	–	–	–	Y	–
Other therapies	SMS-LAR	–	SMS-LAR	–	MMI, SMS-LAR	–	HRT	–	beta-blockers	beta-blockers	Beta-blockers	–	–	Topiramate, TCAs

interf., assay interference; HRT, hormonal replacement; NA, Not available; G, goiter; NG, nodular goiter; AITD, autoimmune thyroiditis; ATA, autonomous thyroid adenoma; SMS-LAR, long-acting-release somatostatin analog; MMI, methimazole; RCC, Renal cell carcinoma; PAF, paroxysmal atrial fibrillation; MGUS, monoclonal gammopathy of undetermined significance; DMD, Duchenne muscular dystrophy; GD, Graves' disease; HT, Hashimoto thyroiditis; TCA, tricyclic antidepressant; Pt variant, pathogenic variant in the THRB gene. *56 days after SMS-LAR, Reference ranges: SHBG:

** Male 10–70;

* *Female 50–144; TSH 0.3–5.1 mU/L; FT4 10–20 pmol/L; FT3 4.2–7.5*.

Patient #2: (i) Referral diagnosis, undetermined IST; The patient presented with multinodular goiter and IST, discovered during investigations for a paroxysmal atrial fibrillation (PAF). PAF was corrected by pharmacologic cardioversion and beta-blockade. An impaired TSH response to i.v. TRH test was found (Table 2). A pituitary MRI showed a right upward convexity without clear evidence of adenoma after gadolinium. (ii) Interventions: A LAR-SMS trial normalized FT3 and FT4 after 3-months, confirming the diagnosis of TSHoma (Table 2). This therapy was well tolerated for 2 years, but then an attack of biliary colic necessitated discontinuation of LAR-SMS and treatment with methimazole (MMI) instead, prior to cholecystectomy. As expected, TSH increased markedly and after 6 months of MMI, pituitary MRI scan now visualized an 8 mm right sided lesion consistent with microadenoma. Likely, MMI had promoted the growth of a microadenoma which was then uncovered. The patient underwent trans-sphenoidal surgery (TSS) and histological examination confirmed a TSHoma. Postsurgically, TSH was persistently suppressed and unresponsive to TRH stimulation, but with FT3 and FT4 levels remained normal. Thyroid scintigraphy revealed a hyperfunctioning right nodule with suppressed contralateral lobe uptake. Following subsequent thyroidectomy and L-T4 replacement, euthyroid status was finally achieved. (iii) Ultimate diagnosis, TSHoma associated with autonomous thyroid nodule.

Patient #3: (i) Referral diagnosis, TSHoma. This patient had Duchenne muscular dystrophy (DMD) of childhood onset. Biochemical evaluation for low bone density showed discrepant TFTs, confirmed on different assay platforms. Although TPO-Ab and Tg-Ab were negative, thyroid ultrasonography showed heterogeneous echotexture with pseudonodules. The TRH test showed a normal increase of the TSH after TRH stimulation, and MRI scan showed a 3 mm left-sided pituitary lesion. Sequencing of *THRB* and ALB did not identify any pathogenic variants. With T3 administration being contraindicated due to DMD, a LAR-SMS trial (20 mg/day) was undertaken, with normalization of FT4 and FT3. Twelve months later FT4 became elevated again, while FT3 levels were within the normal range. LAR-SMS dosage was increased up to 30 mg/28 days without improvement. (ii) Interventions: Thyroid function was rechecked using a two-step assay platform and found to be normal (FT4 14.8 pmol/L, RR 10–20, total T4 (TT4) 118.9 nmol/l, RR 69.0–141.0 nmol/L). Thyroxine binding globulin (TBG) levels were low (13 μg/ml RR 14–31), probably due to corticosteroid treatment. In addition, he was also taking biotin as a supportive therapy for DMD, which might have caused interference with avidin/streptavidin-based assay methods (24). (iii) Ultimate diagnosis, biotin assay interference.

Patient #4: (i) Referral diagnosis, RTHβ. In this patient, discrepant TFTs (TSH 1.67 μIU/ml RR 0.28–4.3, FT3 15.8 pmol/l RR 2.9–7; FT4 91.6 pmol/l RR 10–20), were discovered during investigation for lipodystrophy, hyperinsulinemia, and weakness. TSH increased 5-fold following TRH stimulation and suppressed after T3 administration. A 3 mm pituitary lesion was visualized on MRI scan. LAR-SMS administration for 3 months resulted in reduction of FT3 and FT4 (29% FT4; 26% FT3) levels. TPO-Ab and Tg-Ab were both negative, and thyroid ultrasound scan showed a slightly heterogeneous pattern. Sequencing of *THRB* and ALB in the patient did not identify any pathogenic variant. (ii) Intervention: TFTs were rechecked using a two-step assay platform and found to be normal (FT4 13.2 pmol/l RR 10–20 and TT4 93.9 nmol/l RR 69.0–141.0). Thyroxine binding globulin (TBG) levels were normal (20.6 nmol/L RR 14-31); (iii) Ultimate diagnosis, assay interference, but its cause remains unclear.

Patient #5: (i) Referral diagnosis, assay interference. The patient first presented with panic disorder, but at that time thyroid function was normal. One year later, thyroid function tests consistent with IST were noted but not confirmed on a second sample and the patient was discharged with no further investigation. Five years later, a relapse of psychiatric symptoms was associated with recurrent discrepant TFTs. Thyroid ultrasound scan showed a pattern suggestive of autoimmune thyroid disease. Assay interference due to macro-TSH was excluded by PEG precipitation of serum and column chromatography. (ii) Interventions: TSH levels did not change following TRH stimulation or T3 suppression. MRI scan showed no pituitary lesion. LAR-SMS treatment for 3 months resulted in reduction of FT4 and FT3, consistent with an underlying TSHoma (Table 2). However, 20 days after the last LAR-SMS injection thyrotoxic symptoms worsened, now associated with suppressed TSH and positive TRAb level, leading to diagnosis of Graves' disease (GD) in combination with TSHoma. The patient had no features of Graves' orbitopathy. Following MMI treatment FT4 and FT3 normalized, but eventually FT3 and FT4 rose again with concurrent normal TSH levels after MMI discontinuation. (iii) Ultimate diagnosis, TSHoma.

Patient #6: (i) Referral diagnosis, TSHoma. The patient was diagnosed with IST and MMI treated for a few months. After discontinuation of thionamide, the patient underwent MRI scan, visualizing a pituitary lesion compatible with microadenoma. Although TSH levels rose normally after TRH stimulation (Table 2), the patient proceeded to TSS without further investigation. Histological examination of removed tissue showed no evidence of adenoma. Furthermore, IST was unchanged after surgery and she was referred to us in 1997. (ii) Interventions: We assessed thyroid function in first-degree relatives, documenting IST in three of cases. Sequencing revealed the heterozygous R429Q *THRB* mutation in all of them. (iii) Ultimate diagnosis, RTHβ.

Patient #7: (i) Referral diagnosis, RTHβ. The patient presented with tremor and weight loss. Thyroid function tests showed IST with positive anti-thyroid autoantibodies. Thyroid ultrasound showed a multinodular goiter and FNAB of a 33 mm nodule excluded malignancy. A TRH test showed a normal rise in TSH and the pituitary MRI scan was negative. The patient was classified as a case of non-TRβ-mediated RTH and monitored. Over the next 2 years, baseline TSH, FT3, and FT4 levels increased and the peak TSH response following TRH stimulation showed a diminished increment compared to baseline (4 and 9 fold increase, respectively). (ii) Interventions: Suppression of TSH following T3 administration was blunted (from 5.9 to 2.7 μUI/ml) but still responsive to TRH (TSH peak 19.7 μUI/ml). Treatment with LAR-SMS normalized FT4 and FT3 levels. Repeat MRI scan now showed a small pituitary lesion and the patient underwent TSS and then developed hypopituitarism. Histopathology confirmed the presence of a TSHoma. (iii) Ultimate diagnosis, TSHoma.

Patient #8: (i) Referral diagnosis, TSHoma. The patient was diagnosed with autoimmune hypothyroidism on the basis of raised TSH (5.06 μUI/ml), normal free thyroid hormones (FT4 12 pg/ml RR 7.5–17.5, FT3 3.7 pg/ml 2.3–4.2), and positive anti-TPO antibodies. Due to concurrent mild hyperprolactinemia (PRL 57 ng/ml; RR 3–20), the patient underwent pituitary MRI scan which showed a normal gland. L-T4 treatment promptly normalized thyroid function and PRL levels. Five years later, IST became apparent and persisted despite discontinuation of L-T4. This biochemical picture was confirmed in different laboratories and mild symptoms of hyperthyroidism were noted, raising suspicion of a TSHoma. However, dynamic tests were suggestive of RTHβ (Table 2) and a further MRI scan was negative. (ii) Intervention: When tested using a two-step assay, patient's thyroid function was found to be normal (TSH 3.03 μUI/ml, FT4 16.1 pmol/l, FT3 7.2 pmol/l, and TT4 levels 92.9 nmol/l, RR 69–141 nmol/L). (iii) Ultimate diagnosis, assay interference.

Patient #9 (i) Referral diagnosis, RTHβ. She presented with discrepant TFTs and this was confirmed in different laboratories. Tg-Ab and TPO-Ab were negative and she had a normal thyroid gland on ultrasonography. Pituitary MRI scan showed a 7 mm left-sided lesion with deviation of the pituitary stalk. Mild osteopenia and tachycardia, treated successfully with beta blockade, was documented. The TSH response to TRH stimulation and T3 suppression was normal and *THRB* sequencing was negative, such that the patient was classified as “non-TRβ-RTH” with a non-functioning pituitary incidentaloma, and monitored. (ii) Intervention: When retested using a two-step assay platform, FT4 (13.9 pmol/L), FT3 (4.5 pmol/L), and TT4 (111 nmol/L RR 69.0–141.0 nmol/L) levels were found to be normal. (iii) Ultimate diagnosis, assay interference.

Patient #10 (i) Referral diagnosis, RTHβ. In this patient IST was identified during investigation for PAF. The biochemical pattern was confirmed in different laboratories and the TSH response to TRH stimulation was normal while T3 suppression test was contraindicated. MRI scan showed a normal pituitary gland, whilst ultrasound showed a slightly hypoechoic thyroid and 99-Tc scan thyroid uptake was normal. Anti Tg-Ab levels were negative and anti TPO-Ab positive. (ii) Intervention: *THRB* and ALB sequencing did not identify any variants. High TT4 (213.1 nmol/L RR 69.0–141.0 nmol/L) and a normal FT4 levels by a two step-assay (10.4 pmol/L) were documented, suggesting interference due to antibodies (5). At serum electrophoresis we found two monoclonal IgA K-chain components (0.50 g/dL and <0.30 g/dL, respectively) which may explain this interference (25–27). (iii) Ultimate diagnosis, assay interference.

Patient #11: (i) referral diagnosis, RTHβ. Discrepant TFTs were discovered during investigation for weight loss and thyrotoxic symptoms. The discrepant pattern of TFTs was confirmed in a second laboratory, the TSH response to TRH stimulation was normal, but the patient declined a T3 suppression test. MRI showed a normal pituitary gland and the thyroid gland had a normal ultrasonographic appearance. Anti Tg-Ab and anti TPO-Ab levels were negative. (ii) Interventions: *THRB* and ALB sequencing was normal. When retested using a two-step assay platform, we found normal TT4 (108.6 nmol/L RR 69.0–141.0 nmol/L) and FT4 (12.1 pmol/l) levels. (iii) Ultimate diagnosis, assay interference.

Patient #12: (i) referral diagnosis, TSHoma, and Graves' disease. This patient presented with hyperhidrosis and tachycardia and discrepant TFTs (TSH 0.57 and 0.44 μUI/ml, FT4 62.5, and >100 pmol/l, FT3 11.5, and 12 pmol/l). The patient had a multinodular thyroid gland on ultrasonography along with high TRAb levels (21.6 UI/ml RR < 1.6) leading to the suspect of a TSHoma associated with Graves' disease. (ii) Interventions: SHBG were inappropriately normal for hyperthyroidism (77 pmol/l RR 50–144), the 99mTc uptake was normal and not clear signs of thyroxicosis were found at clinical assessment. At serum electrophoresis a significant monoclonal IgM peak (1.36 g/dl) was found. Patient's thyroid function reassessed by another one-step platform (Beckman) resulted in normal FT3 and slightly elevated FT4 levels (TSH 0.92 RR 0.34–5.6 μUI/ml; FT3 3.85 pg/ml RR 2.5-3.9; FT4 1.33 ng/dl RR 0.61–1.12) while a two-steps assay showed normal free and total TH (FT4 12.9 pmol/l RR 10–20, TT4 94.9 nmol/L RR 69–141, TT3 1.5 nmol/L RR 1.3–2.5). Several other endocrine and not endocrine abnormalities were found which were all reversible after 20% PEG precipitation or serial serum dilution of the serum. Six sandwich immunoassays (TSH, LH, FSH, ACTH, PTH, and PRO-BNP) were underestimated, while competitive assays (FT4, FT3, and TRAb) were overestimated, as previously reported in patients with paraproteins (25, 27). (iii) Ultimate diagnosis: assay interference.

Patient #13: (i) Referral diagnosis, thyroiditis. The patient presented with slightly high FT3 levels (8 pmol/l) associated with a normal TSH and FT4 (2.75 μUI/ml and 17.6 pmol/l, respectively) with mild thyrotoxic symptoms. A thyroid scintigraphy showed an area of reduced uptake in the right lobe without US detectable nodules which was attributed to a “silent” thyroiditis. No further investigation were performed for 1 year, although persistent high FT3 levels. (ii) interventions: TRH test and T3 suppression were performed and consistent with a TSHoma (Table 2). A 3 mm pituitary lesion was found by MRI. After TSS, baseline and TFTs, normalized and TSH retained a normal response to TRH and T3 administration in the following 10 years. Six months prior to TSS a left 4 cm thyroid cyst was noted which was treated with percutaneous ethanol injection. (iii) Ultimate diagnosis, TSHoma.

Patient #14: (i) Referral diagnosis: drug-induced artifact. This patient presented with isolated high FT4 levels (TSH 1.58 μUI/ml; FT3 4.0 pg/ml RR 2–5; 22.8 pg/ml RR 8–17), and hyperprolactinemia (53.4 and 113.5 ng/ml RR 5–20) during treatment with tricyclic antidepressant (clomipramine), antipsychotic agents (olanzapine and risperidone), and topiramate for a psychiatric bipolar disorder. At MRI a 3 mm cyst of the pars intermedia of the pituitary was found. TPO-Ab and Tg-Ab were negative although a US thyroid pattern suggestive of AITD; a normal TSH response to TRH was found. (ii) Interventions: 2 years later, FT4 levels persisted elevated despite topiramate withdrawal, while PRL normalized when cabergoline was discontinued during aripiprazole treatment. *THRB* sequencing showed the p.M310V variant of the gene. (iii) Ultimate diagnosis: RTHβ.

## Discussion

We reviewed baseline and dynamic testing in patients suspected with IST, and present challenging case histories from 14 patients in whom the correct diagnosis was either missed or delayed due to misinterpretation of clinical data.

**Figure 4:**
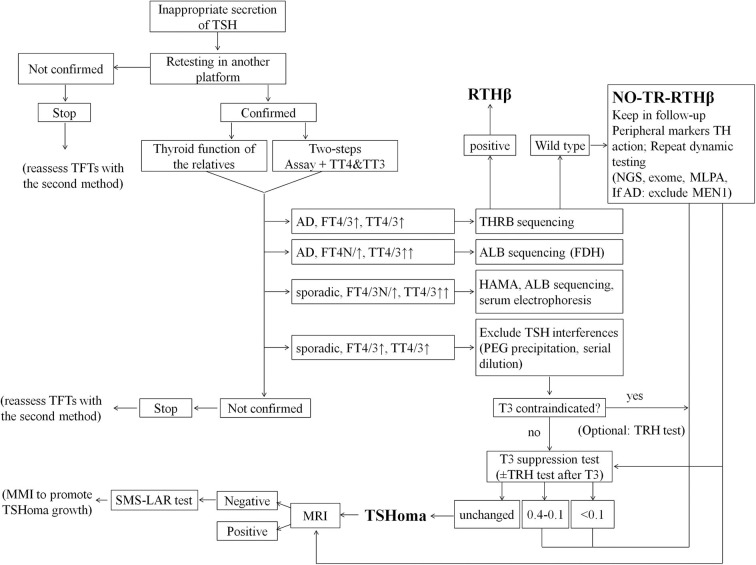
Flowchart for more effective differential diagnosis of inappropriate TSH secretion. This flow-chart represents our proposed cost-effective work up for the differential diagnosis of inappropriate TSH secretion. Final diagnoses are indicated in bold; optional tests are indicated in parentheses. AD, autosomal dominant; ALB, albumin gene; FDH, Familial dysalbuminemic hyperthyroxinemia (FDH); HAMA, Human anti-mouse antibodies; MEN1, Multiple Endocrine Neoplasia type 1; MLPA, Multiplex Ligation Probe Amplification; MMI, methimazole; NGS, next generation sequence; PEG, polyethylene glycol; SMS-LAR, long-acting-release somatostatin analog; TFTs, thyroid function tests; TH, thyroid hormones; TT4, total T4; TT3, total T3.

Reflecting our conclusions and experience we propose a revised diagnostic flowchart (Figure 4) which encompasses and amplifies upon published guidelines (1). In particular, we have included a detailed description of the differential diagnosis of interferences causing artifacts in assays of TFTs. Moreover, now we give more emphasis to T3 suppression than TRH test or peripheral markers of TH action, and we excluded from the investigations the αGSU because of its unavailability in routine settings worldwide. We also provide specific cut-offs for the interpretation of T3 suppression test. In addition, we have indicated how the diagnosis of non-TR-RTHβ can be reached by a combination of dynamic tests and peripheral markers of TH action. Finally, we propose the administration of anti-thyroid drugs in selected cases of microTSHoma under the limits of MRI detection but diagnosed by SMS-LAR test. This would accelerate the growth of an underlying lesion within some months and then allow the surgical approach.

Our observations indicate assay interference as the main source of error that is still too often underestimated. Unfortunately, the widespread use of high-throughput platforms based on one-step assays has increased the frequency of assay artifact, due to interference from biotin, circulating heterophilic antibodies or abnormal binding proteins for example. Automated, one-step, measurement technologies are reproducible and cost efficient, favoring their use over more robust and bespoke two-step methods (where the labeled TH is added only after incubation of serum with the anti-TH antibody in solid phase and the washing step removing all potential interfering substances), particularly when assay services are centralized in laboratories following competitive tendering processes. However, the lack of robust assays in referral centers investigating difficult cases using complex investigation pathways may paradoxically increase the costs of diagnosis. Thus, in this patient cohort, cases of apparently discrepant TFTs due to assay interference were investigated with pituitary imaging, dynamic endocrine testing, genetic analyses and LAR-SMS test (Patient #3 and #4). In addition, patient 4 underwent LAR-SMS treatment for further 15 months. According to the schedule of charges in the National Health System in Italy, these procedures and treatments caused an estimated cost >35,000 € (~37,000 USD), not taking into account the indirect costs (e.g., working hours lost due to unnecessary investigation). Conversely, the cost of thyroid function testing for these six patients using a two-step assay would have been lower than 300 €.

In addition, we recommend retesting TFTs periodically and with different assays when the source of the assay interference is not clearly evident. Interestingly, in three patients with genuine TSHomas (#1 and #13) or RTHβ (#14) the presence of an isolated high FT4/FT3 delayed the correct diagnosis being attributed to an artifact.

Previous literature data suggest that several variables (gender, thyroid autoimmunity, and other thyroid dysfunctions) can influence the response to dynamic endocrine testing—particularly the TRH stimulation test (1, 18, 28). Indeed, in order to improve its specificity, some authors have suggested performing the TRH test following the administration of L-T3 for 3 days (14). Such testing would show complete suppression of the TSH response to TRH in normal individuals, partial suppression of TSH response in patients with RTHβ, and no suppression in TSHoma cases. However, in our cohort, two genetically-proven RTHβ patients showed TSH levels between 0.5 and 0.6 even 10 days after T3 administration, raising doubts about the ability of T3 administration for a shorter period of 3 days to suppress TSH levels effectively.

Patient #7 exhibited a 9-fold rise in TSH after TRH, possibly due to coexisting Hashimoto's thyroiditis; due to absence of features of a pituitary tumor she was incorrectly diagnosed as “non-TRβ-RTH.” Ultimately, reevaluation of thyroid status in this patient proved to be the most correct strategy. As previously reported (28) the diagnosis of a TSHoma can be delayed if initial investigations are suggestive of RTHβ. In our patient and in the two patients of Macchia et al. (28), the administration of LAR-SMS clarified the diagnosis. Conversely, in the differential diagnosis of discrepant TFTs, concomitant primary hyperthyroidism can cause a blunted response to TRH. For example, one RTHβ patient with an underlying “warm” thyroid nodule showed a blunted TSH response (baseline TSH 0.26; peak TSH 3.2 μUI/ml) at TRH test. Our observations suggest that in patients with coincident primary thyroid disease (e.g., autoimmune thyroid disease or multinodular goiter), the T3 suppression test alone, or in combination with response to LAR-SMS administration, has the highest diagnostic specificity for TSHoma, with the TRH stimulation test being potentially misleading. The T3 suppression test is usually safe and absolute contraindications (severe cardiac disorders, atrial fibrillation, untreated sinus tachycardia, uncorrected adrenal cortical insufficiency) are limited. The test is usually avoided in the elderly as liothyronine may exacerbate occult underlying cardiac problems such as ischemic heart disease, angina pectoris, arrhythmia, or congestive heart failure.

Another observation is the variable predictive value of negative or positive response to dynamic testing. In other words, a subnormal TSH response following TRH stimulation strongly suggests a diagnosis of TSHoma, but only when thyroid autonomy has been ruled out. Conversely, complete suppression of TSH after T3 administration excludes TSHoma, whilst partial or incomplete TSH suppression, implying mutant receptor insensitivity to thyroid hormone, favors a diagnosis of RTHβ (Figure 2).

In patient #6 an excessively stringent interpretation of MRI scan result was the main reason for misdiagnosising TSHoma, rather than correct identification of RTHβ. Results of the pituitary MRI scan should always be interpreted with caution, as pituitary incidentalomas are frequent in the general population, and so would be expected to arise as frequently in patients with spurious hyperthyroxinemia due to assay interference (e.g., patients #3, #4, and #9). Conversely, micro-TSHomas may fail to be visualized in pituitary MRIs (e.g., patients #2, #5, and #7). In some cases with an initial negative pituitary MRI scan (e.g., patient #2), a short course of anti-thyroid drug treatment can not only control hyperthyroidism, but also promote an increase in size of an underlying microadenoma which is then visualized on further imaging. In addition, the case history of patient #2 illustrates the fact that permanently suppressed TSH levels following pituitary surgery, in combination with detectable TH levels while not on thyroxine therapy, may denote the presence of concomitant, but previously hidden autonomous thyroid nodules.

Patient #6 illustrates the high specificity of finding abnormal thyroid function tests in first-degree relatives; thus, in nearly 80% of RTHβ cases, testing family members can avoid misdiagnosis and unnecessary further investigation (27). Surprisingly, in 30% of the RTHβ cases included in this study the patients' relative were not screened before referral, although the probability of having RTHβ (incidence 1:20.000–40.000 newborns) (4) is higher than that of having a TSHoma (prevalence <1 per 1 million inhabitants) (2, 3).

Interestingly, in two patients (#2 and #5), persistent inhibition of TSH secretion from normal thyrotropes following surgical removal of the TSHoma or its inhibition with LAR-SMS has unmasked underlying, concomitant primary hyperthyroidism. This is a possibility that should be considered in order to avoid misinterpreting results to indicate either central hypothyroidism following TSS (e.g., patient #2) or unresponsiveness to LAR-SMS (e.g., patient #5).

Due to physiological decrease in FT4 and FT3 levels in second and third trimester, pregnancy may mask diagnosis of IST. Thus, in one patient included in this series, failure to reassess thyroid status after delivery, caused delay in the diagnosis.

The determination of α-GSU serum levels appears to be of limited utility in the differential diagnosis of IST. Thus, a high α-GSU/TSH molar ratio was documented in five post-menopausal women with genetically-proven RTHβ, whereas 3/14 patients with micro-TSHoma exhibited a normal α-GSU/TSH molar ratio. Our data also suggests that the absolute magnitude of αGSU is not sensitive enough for differential diagnosis of IST, as levels were normal in several patients with a micro-TSHoma. As suggested by Socin et al. (16) TSHomas are now increasingly diagnosed as microadenomas (<1 cm), and consequently the percentage of patients with raised αGSU levels is lower than previously reported (29, 30).

The 14 patients included in this series experienced a diagnostic delay of >2 years following the estimated onset of clinical manifestations of thyrotoxicosis. Interestingly, this interval is similar to that reported in Cushing' disease (31). Finally, our case series shows that self-reported symptoms and signs in patients can be misleading, since some individuals with assay interference exhibited features of apparent thyrotoxicosis.

The whole of these findings has to be seen in light of its retrospective nature. However, given the rarity of the clinical conditions, appropriate powered prospective study should require several decades to enroll enough cases.

In conclusion, we have retrospectively surveyed, a large series of discrepant TFT patients and controls, and described 14 patient case histories in detail, illustrating the main pitfalls in making a correct diagnosis. In selected cases with apparent “non-TRβ-RTH,” a short course of LAR-SMS administration or the T3-suppression test represented the best tools to correctly diagnose TSHomas with paradoxical responses to TRH stimulation. Recognition of assay interference is a mandatory and cost-effective first-line approach in order to avoid further unnecessary investigation and potentially harmful therapy, as the TSH response to TRH stimulation or T3 suppression cannot distinguish between raised thyroid hormones due to interference in FT4 and FT3 assays or RTHβ.

A multistep investigation protocol, with careful interpretation of all test results including molecular genetic studies, appears to be the best strategy for correct, early differential diagnosis of discrepant TFTs—even in those cases in which the cause appears initially obvious (Figure 4).

## Data Availability Statement

The raw data supporting the conclusions of this article will be made available by the authors, without undue reservation.

## Ethics Statement

The studies involving human participants were reviewed and approved by EC, Istituto Auxologico Italiano IRCCS. The patients/participants provided their written informed consent to participate in this study.

## Author Contributions

IC designed the study, analyzed the data, and wrote the draft of the manuscript. DC, CC, FO, and GG collected the data. CM performed some serological assays. LP, PB-P, CM, and KC reviewed and revised the manuscript. The Authors acknowledge the referral of one patient from Prof. Renato Pasquali, (Bologna) who recently passed away. All authors contributed to the article and approved the submitted version.

## Conflict of Interest

The authors declare that the research was conducted in the absence of any commercial or financial relationships that could be construed as a potential conflict of interest. The reviewer EF declared a past co-authorship with one of the authors LP to the handling editor.
